# Analysis of the expression of FAP, Caveolin1, and CPXM2 and prognostic significance in gastric cancer

**DOI:** 10.3389/fonc.2026.1716536

**Published:** 2026-04-13

**Authors:** Lijie Zhou, Huihu He, Tie Zhao, Wang Du, Guoping Lu, Lingjun Geng

**Affiliations:** 1 Department of Clinical Medical Laboratory, The Fuyang Affiliated Hospital of Anhui Medical University, Anhui, Fuyang, China; 2 Department of General Surgery, The Fuyang Affiliated Hospital of Anhui Medical University, Anhui, Fuyang, China; 3 Center for Genomic Medicine, Graduate School of Medicine, Kyoto University, Kyoto, Japan

**Keywords:** cancer associated fibroblasts, CAV1, CPXM2, FAP, gastric cancer, prognosis

## Abstract

**Background:**

This study sought to examine the expression levels and clinical relevance of fibroblast activation protein alpha (FAP), caveolin-1 (CAV1), and carboxypeptidase X member 2 (CPXM2) in gastric cancer (GC) and adjacent tissues.

**Methods:**

Multiplex immunofluorescence (mIF) was performed on tissue microarrays to evaluate the expression of FAP, CAV1, CPXM2, and cytokeratin (CK) in GC specimens. Correlations among these markers and their associations with clinicopathological features and survival outcomes were analyzed.

**Results:**

This investigation discovered that CAV1 was predominantly expressed in stromal cells, whereas CPXM2 was expressed in both epithelial and stromal cells. The number and proportion of FAP^+^ and CAV1^+^ cells were significantly higher in GC tissues than in adjacent tissues, whereas CPXM2^+^ cells were significantly reduced, and there was no significant difference in CK^+^ cells. We employed CK to differentiate epithelial cells from stromal cells and found that there was no notable difference in the counts and proportion of CPXM2^+^ cells between epithelial and stromal cells in GC tissues. Further research revealed that, in comparison with adjacent tissues, the number and proportion of CPXM2^+^ cells in epithelial cells of GC tissues were significantly reduced. FAP was utilized as a marker for cancer associated fibroblasts (CAFs) in subsequent analyses. Additional investigations demonstrated that the counts and proportion of CAV1^+^ cells in CAFs were notably increased in GC tissues, while there was no significant difference in CPXM2^+^ cells. A strong positive correlation was observed between CAV1^+^ and CPXM2^+^ cells in GC tissues. Correlation analysis of clinicopathological parameters indicated that the proportion of FAP^+^ cells correlated with AJCC stage and tumor invasion depth; CPXM2^+^ cells correlated with tumor location; Epithelial CPXM2^+^ cells were associated with the patient’s age. CAFs with CAV1^+^ and CPXM2^-^CAV1^+^ cells were linked to the AJCC stage, invasion depth, and tumor location. Higher proportions of FAP^+^, FAP^+^CAV1^+^, and FAP^+^CPXM2^-^CAV1^+^ cells were associated with poorer overall survival (OS).

**Conclusions:**

These findings suggest that CAV1 and CPXM2 are differentially expressed in GC and associated with patient prognosis, highlighting them as potential candidates for further functional investigation in the context of GC progression.

## 1 Introduction

Over the past few decades, despite the consistent decline in gastric cancer occurrences, the mortality rate of GC still remains relatively high (1). For advanced gastric cancer, sequential lines of chemotherapy are employed; nevertheless, it remains challenging to extend the median survival (2). Besides surgical resection and conventional radiotherapy, chemotherapy and targeted immunotherapy have emerged in recent years. Given its remarkable therapeutic efficacy and minimal side effects, immunotherapy has become an additional approach for treating GC (3). However, immunotherapy does not yield effective results for all gastric cancer patients. Thus, it is imperative to identify current prognostic and therapeutic targets and subsequently implement personalized treatment strategies for different individuals. The tumor microenvironment (TME) includes malignant cells along with native and infiltrating host cells, notably CAFs and immune cells (4). Within the TME, aside from immune cells, whether additional stromal cells, like CAFs, possess the potential for immunotherapy warrants further investigation.

Fibroblasts are among the earliest cell types recruited to the TME. When they begin to interact with cancer cells, fibroblasts undergo the activation phase. Activated fibroblasts, or CAFs, drive tumor advancement and immune system evasion via various pathways. These include secreting immunomodulatory molecules, engaging in physical interactions with immune cells, and remodeling the extracellular matrix (5). CAFs are typically identified through the expression of a range of characteristic markers, such as α-smooth muscle actin (α-SMA), fibroblast-specific protein 1 (FSP1), and FAP. These markers help distinguish CAFs from the broader population of fibroblasts found throughout the body (6). α-SMA functions not only as a specific marker for CAFs displaying a myofibroblast phenotype. Instead, it is widely recognized as a marker for vascular smooth muscle cells and pericytes (6, 7). FSP1, which is commonly utilized for the identification of fibroblasts, has also been applied as a marker for epithelial cells and macrophages (8, 9). Due to the widespread presence of FAP in the tumor stroma, it has been extensively employed in research as an indicator of activated cancer associated fibroblasts (6) and represents a highly promising therapeutic target against CAFs.

Caveolin-1 (22–24 kDa), encoded by the CAV1 gene on chromosome 7, functions as a scaffold protein. This locus also harbors the CAV2 gene, which expresses caveolin-2. These isoforms assemble into hetero-oligomeric complexes that may incorporate caveolin-3. The collective activity of caveolins generates specialized plasma membrane microdomains with omega-shaped morphology, termed caveolae (10). Nevertheless, Caveolin-1 exhibits context-dependent duality in oncogenesis, functioning paradoxically as both a tumor suppressor and promoter (11). Research findings have indicated that an elevated level of CAV1 expression in tumor cells correlates with adverse clinical outcomes, whereas stromal overexpression associates with improved survival (11). However, further research is required regarding the prognostic relevance of CAV1 expression in GC.

CPXM2, a member of the M14 family, represents a metallocarboxypeptidase (MCP) with limited characterization. Previous research has indicated that CPXM2 is associated with developmental disorders (12, 13) and Alzheimer’s disease (14), but the role of CPXM2 in cancer development or advancement is still uncertain. Recent studies have reported that elevated CPXM2 expression correlates with adverse clinical outcomes in osteosarcoma (15). In gastric cancer, Zhao et al. demonstrated that CPXM2 upregulation is not merely linked to a poor prognosis but also enhances the proliferation and migration capabilities of tumors (16). However, the involvement of CPXM2 in tumorigenesis remains insufficiently characterized, and its functional mechanisms across different types of tumors remain to be fully elucidated.

In this current research, we employed mIF to evaluate the expression levels of CAV1, CPXM2, CK, and CAFs markers, including α-SMA and FAP, in GC tissues. We used the CK index to distinguish epithelial cells from stromal cells and assessed CAV1 and CPXM2 expression within respective cellular compartments. We also sought to investigate the potential correlations among these markers, as well as their associations with clinicopathological parameters and patient prognosis. Additionally, the expression patterns of CAV1 and CPXM2 within CAFs were analyzed concerning fibroblast marker expression, along with their clinical correlations with patient characteristics and outcomes.

## 2 Materials and methods

### 2.1 TMAs

Tissue microarrays were obtained from Outdo Biotech Co., Ltd. (Shanghai, China). One tissue microarray (TMAs, HStm-A180Sur-20) was used to compare the staining effects of CAFs markers FAP and a-SMA, and another tissue microarray (TMAs, HStm-A180Sur-32) was used for the formal experiment. Tissue microarrays (TMAs, HStm-A180Sur-32) were employed. The TMAs comprised 96 GC and 84 non-neoplastic gastric tissue specimens. Each TMA had a core diameter of 1.5 mm, which ensured a larger amount of representative tissues on the TMAs. Tumor staging and differentiation status were evaluated using the TNM classification system (17), with exclusion of patients receiving preoperative chemotherapy or radiotherapy.

### 2.2 Assessing CAV1 expression levels in stromal cells of gastric tissues

Stromal CAV1 expression (including CD8^+^/CD4^+^ T cells, B cells, NK cells, macrophages, endothelial cells, and CAFs) was evaluated using the EPIC algorithm on TCGA-STAD cohorts (Tumor/Normal) via GEPIA2021 (18).

### 2.3 Correlation analysis of CAV1 and CPXM2 in GC

To assess the correlation of CAV1 and CPXM2 expression levels in GC, we employed Spearman’s correlation, which was conducted using TCGA-STAD tumor data through GEPIA2.

### 2.4 Multiplex immunofluorescence

EACRI IHC Core (Providence Cancer Institute, Portland, OR, USA) validated staining protocols, as previously described in prior publications (19). In brief, the tissue microarray (TMA) was incubated at 63 °C for 1 hour, automatically deparaffinized (LEICA ST5020, Leica Biosystems Nussloch GmbH, Nussloch, Germany), and then subjected to antigen retrieval. Commercially available hydrogen peroxidase was then employed to eliminate endogenous peroxidase for 10 minutes. Subsequently, the microarray was subjected to blocking for 10 minutes. Following this, it was incubated with one of the subsequent antibodies for multiplex immunohistochemistry (mIHC) staining over a period of one hour: CAV1 antibody (3267s, 1:1000, Cell Signaling Technology, Danvers, MA, USA), FAP antibody (ab207178, 1:500, Abcam, Cambridge, UK), CPXM2 antibody (abs138862, 1:500, absin, Shanghai, China), CK antibody (PA125, undiluted, abcarta, Suzhou, China), and α-SMA antibody (ab7817, 1:5000, Abcam, Cambridge, UK). After rinsing the slides with TBST, they were treated with the secondary antibody (SM802, ready-to-use, DAKO, Dako Denmark A/S, Glostrup, Denmark) for a 10-minute incubation period. Following antigen retrieval, slides were incubated with Opal dye diluent (Opal 7-color Manual IHC Kit, NEL801001KT, PerkinElmer, Hopkinton, MA, USA) for 10 minutes at room temperature. Antibody complexes were then removed via microwave treatment. Markers were applied sequentially, with this stripping/staining cycle repeated for all targets within the panel. Finally, the nuclei were stained with DAPI for five minutes, and slides were coverslipped using antifade mounting medium.

### 2.5 Image acquisition and quantitative analysis

The Tissue-FAXS system (TissueFAXS Spectra, TissueGnostics GmbH, Vienna, Austria) was employed to conduct panoramic multispectral scanning of slides. Subsequently, StrataQuest analysis software (v7.1.129, TissueGnostics, TissueGnostics GmbH, Vienna, Austria) processed imported data through reference spectral library-based unmixing, generating individual channel fluorescence images. The DAPI channel was employed to demarcate nuclei. Protein-specific fluorescent signals were localized within user-defined radial distances centered on each nucleus, based on staining patterns observed in individual protein channels. Fluorescence intensity thresholds were then established per channel according to staining characteristics (Briefly, at least 10 representative regions were randomly selected from the mIHC images. For each marker, the fluorescence intensity threshold was manually defined based on the theoretical localization pattern of the target antigen together with the evaluation of nonspecific staining and background fluorescence. A threshold that was considered relatively appropriate across all selected regions was then determined and subsequently applied uniformly to all samples. In addition, all threshold settings were reviewed and confirmed by experienced pathologists to minimize subjective bias). This enabled segmentation and quantification of positively stained cells. Concurrently, cells co-expressing two or more markers were enumerated.

### 2.6 Statistical analysis

Statistical analyses utilized SPSS 25.0 (IBM), GraphPad Prism 9.5 (GraphPad Software, San Diego, CA, USA), and R v4.2.2. Group comparisons employed the chi-square test, Welch’s t-test, Mann-Whitney U test, and Wilcoxon signed-rank test. Associations between quantitative levels of each marker (cell counts and percentages) were assessed using Spearman’s rank correlation. Survival distributions were compared via Kaplan-Meier analysis with log-rank testing. The ideal stratification cutoffs for predictive biomarkers were determined via the *surv_cutpoint* tool (R package *survminer*, version 0.5.0). Cox regression models univariate and multivariate facilitated survival analysis, with statistical significance at *p*<0.05 (two-tailed).

## 3 Results

### 3.1 Assessment of CAFs markers expression in GC

**Figure 1:**
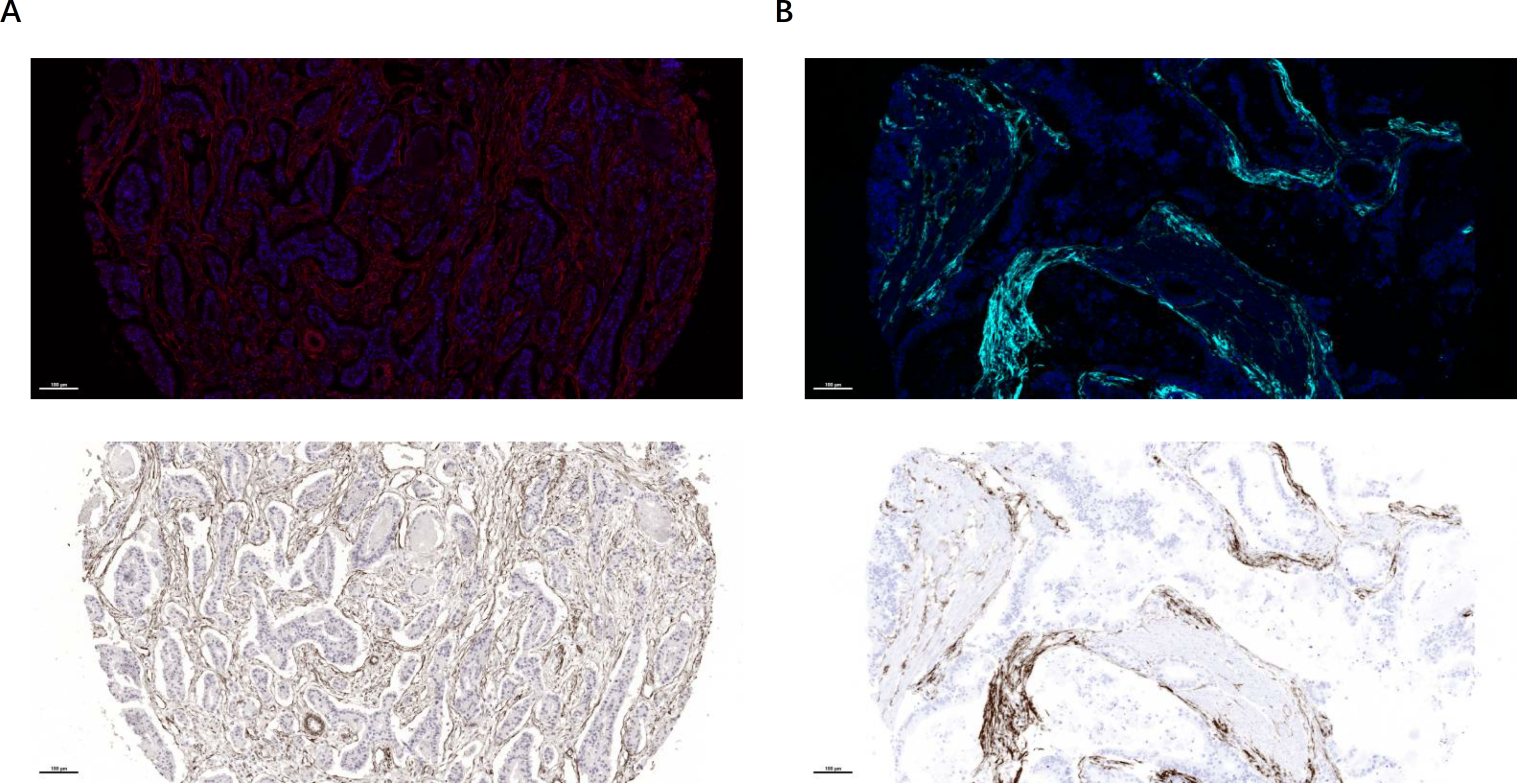
mIF images presented the distribution of CAFs markers within GC tissues. **(A)** Immunofluorescence was employed to detect the expression of α-SMA in CAFs with a myofibroblast phenotype, smooth muscle cells, and pericytes. **(B)** Immunofluorescence was utilized to detect the expression of FAP in CAFs.

Initial evaluation of α-SMA and FAP expression in GC tissue microarrays revealed α-SMA was expressed in CAFs, smooth muscle cells, and pericytes (**Figure 1A**), whereas FAP was specifically localized to CAFs (**Figure 1B**). Therefore, we selected FAP as the specific biomarker of CAFs for subsequent research and analysis.

### 3.2 Expression of FAP, CAV1, CPXM2, and CK in gastric tissues

**Figure 2:**
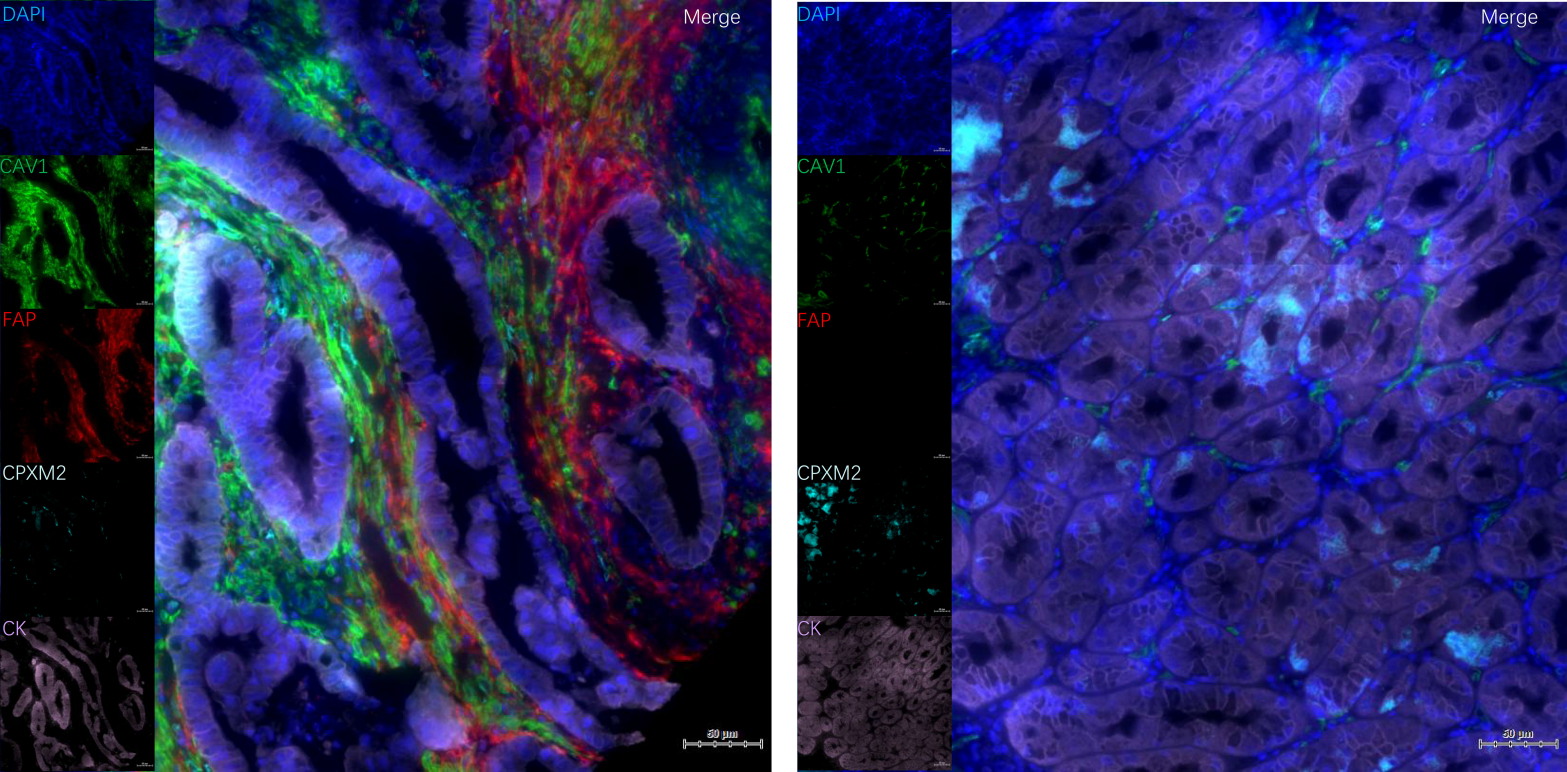
mIF images of gastric tissues with separate channels. Expression of CAV1, FAP, CPXM2, CK and merged images in GC **(A)** and adjacent normal **(B)** tissues.

**Figure 3:**
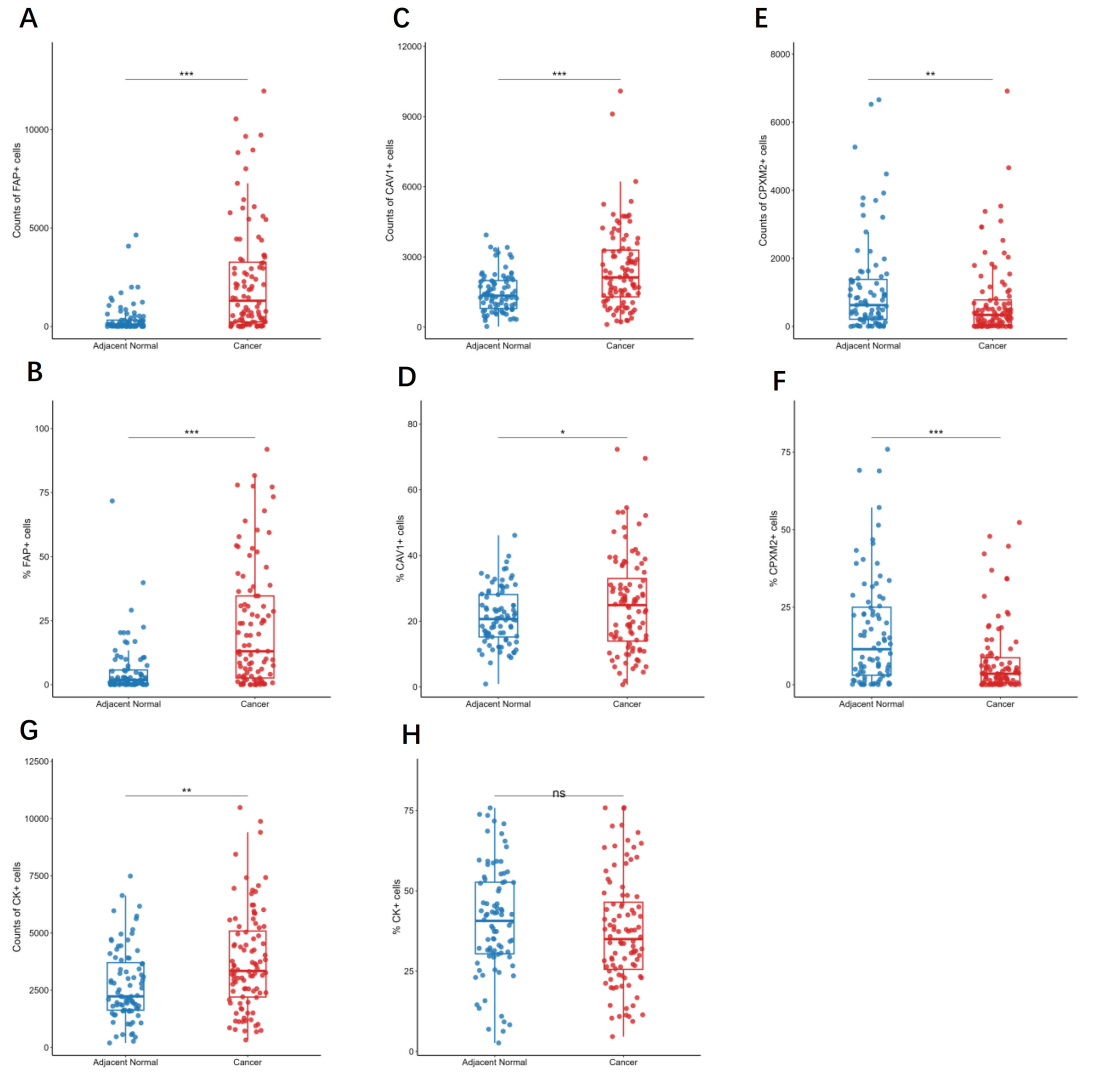
Assessment of FAP, CAV1, CPXM2 and CK expression in gastric tissues. Scatter box plot showed the counts of FAP **(A)**, CAV1 **(C)**, CPXM2 **(E)** and CK **(G)** positive cells and the percentage of FAP **(B)**, CAV1 **(D)**, CPXM2 **(F)** and CK **(H)** positive cells in the specified tissue samples. Group comparisons were evaluated via Welch’s t-test **(D, H)** and Mann-Whitney U test. *p < 0.05, ** *p* < 0.01, *** *p* < 0.001, ns, not significant.

Representative mIF images were shown in **Figure 2**. Compared to adjacent tissues, GC tissues exhibited significantly higher counts and proportions of FAP^+^ cells (both *p* < 0.001; **Figures 3A, B**). CAV1^+^ cells were also increased in GC tissues (*p* < 0.001 for count, *p* = 0.045 for percentage; **Figures 3C, D**). In contrast, CPXM2^+^ cells were significantly decreased (*p* = 0.01 for count, *p* < 0.001 for percentage; **Figures 3E, F**). The count of CK^+^ cells was higher in GC (*p* = 0.001; **Figure 3G**), but the proportion of CK^+^ cells did not differ significantly (*p* = 0.203; **Figure 3H**).

**Figure 4:**
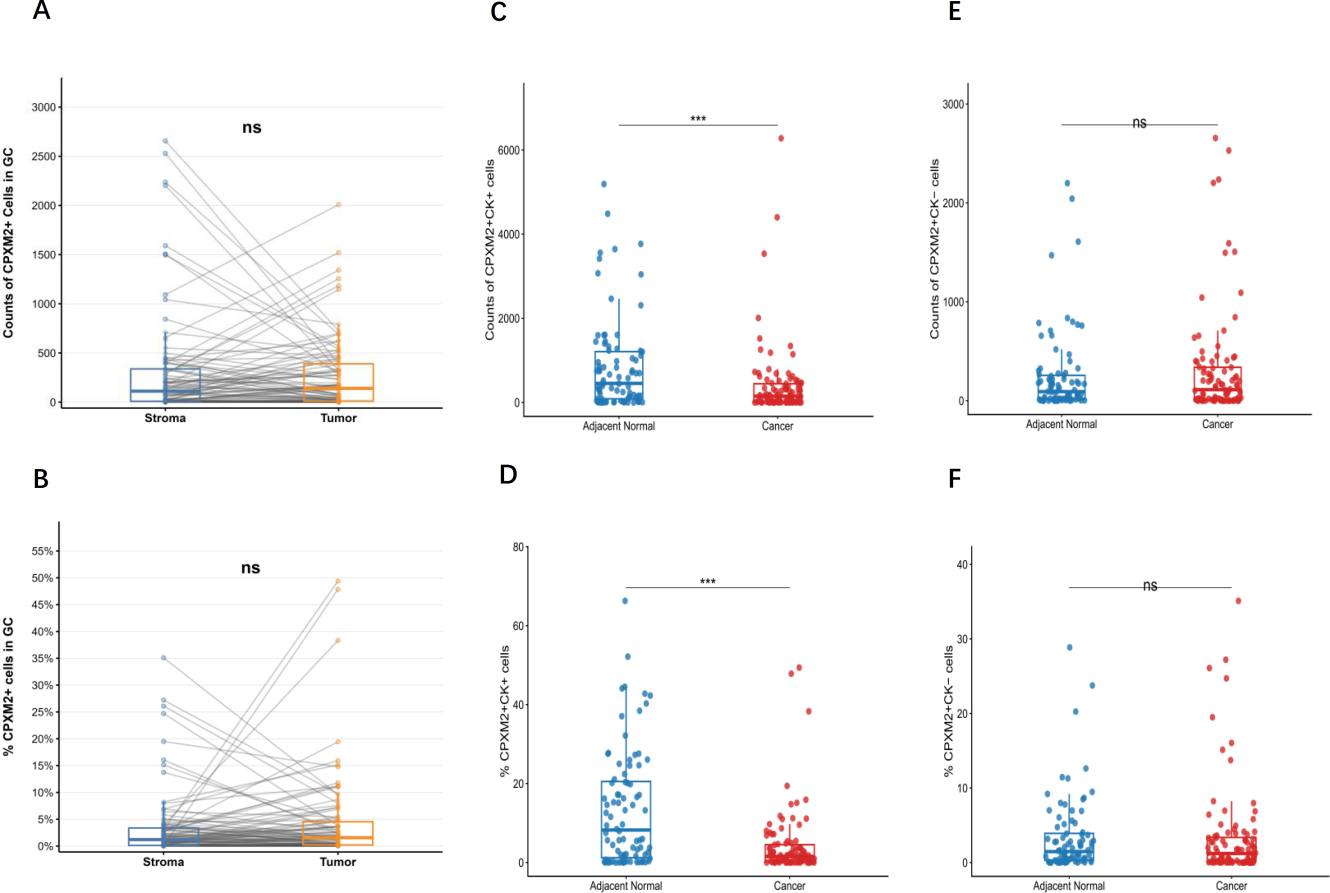
Evaluation of CPXM2 expression in gastric tissues. Paired boxplots showed the counts of CPXM2^+^ **(A)** cells and the proportion of CPXM2^+^ **(B)** cells in the specified tissue samples. Scatter box plot showed the counts of CPXM2^+^CK^+^ **(C)** and CPXM2^+^CK^-^ **(E)** cells and the percentage of CPXM2^+^CK^+^ **(D)** and CPXM2^+^CK^-^ **(F)** cells in the specified tissue samples. Group comparisons were evaluated via Wilcoxon rank test **(A, B)** and Mann-Whitney U test. *p < 0.05, ** *p* < 0.01, *** *p* < 0.001, ns, not significant.

Using the CK index to distinguish epithelial from stromal cells, we assessed CAV1 and CPXM2 expression in these compartments. CAV1 was predominantly expressed in stromal cells, whereas CPXM2 was expressed in both epithelial and stromal cells. No significant differences were observed in the counts or percentage of CPXM2^+^ cells between GC tissues and stroma (both *p* > 0.05; **Figures 4A, B**). However, both the number and proportion of CPXM2^+^CK^+^ cells were significantly lower in GC tissues than in adjacent normal tissues (*p* < 0.001; **Figures 4C, D**). In contrast, neither the counts nor the percentage of CPXM2^+^CK^-^ cells differed significantly between GC and adjacent tissues (both *p* > 0.05; **Figures 4E, F**).

### 3.3 Expression of CAV1 and CPXM2 in CAFs

**Figure 5:**
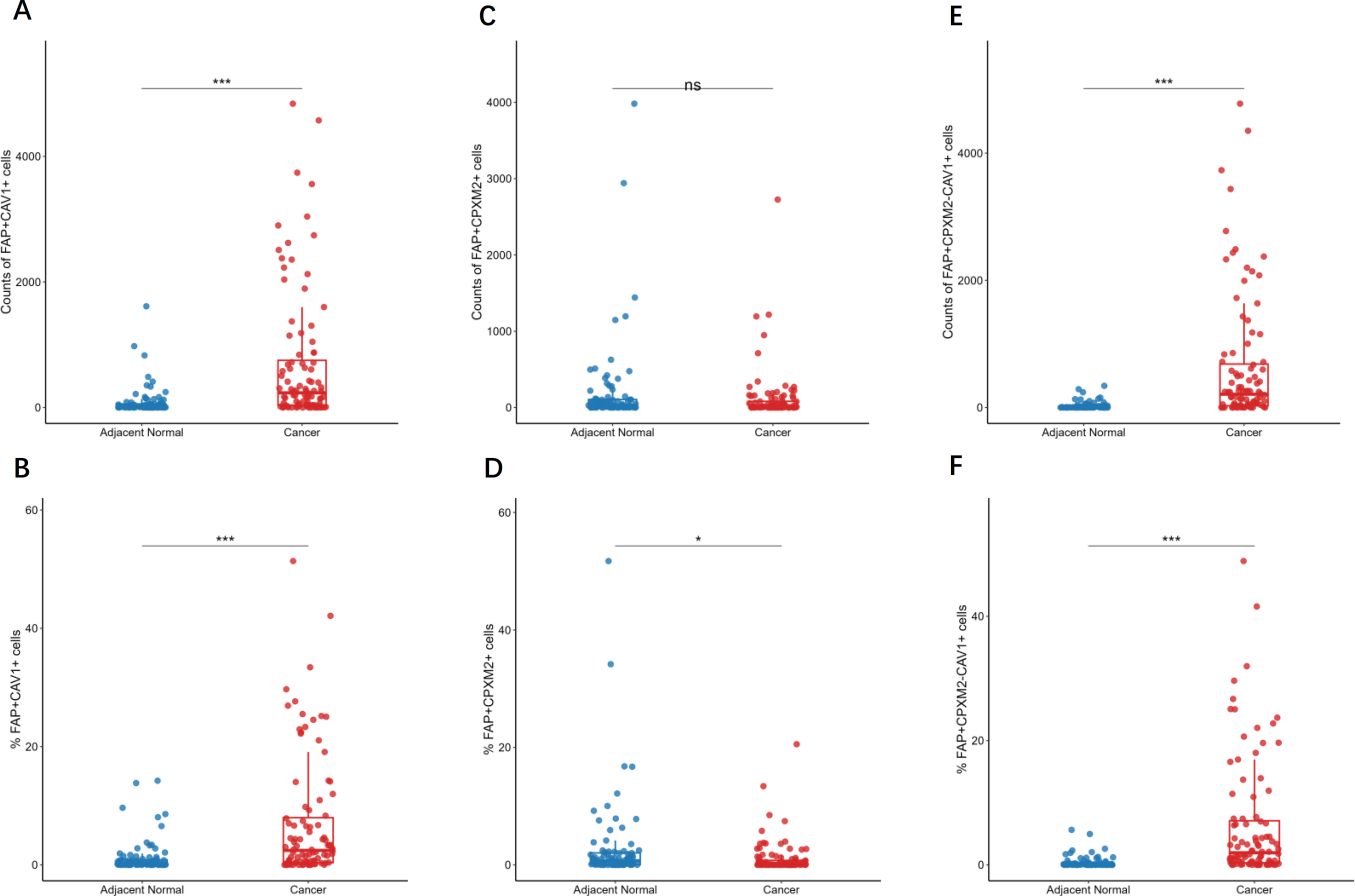
Evaluation of CAV1 and CPXM2 expression in CAFs. Scatter box plot showed the counts of FAP^+^CAV1^+^ **(A)**, FAP^+^CPXM2^+^ **(C)** and FAP^+^CPXM2^-^CAV1^+^ **(E)** cells and the percentage of FAP^+^CAV1^+^ **(B)**, FAP^+^CPXM2^+^ **(D)** and FAP^+^CPXM2^-^CAV1^+^ **(F)** cells in the specified tissue samples. The Mann-Whitney U test was employed to analyze the statistical tests among groups. *p < 0.05, ** *p* < 0.01, *** *p* < 0.001, ns, not significant.

We then investigated the expression of CAV1 and CPXM2 in CAFs. The proportion and counts of FAP^+^CAV1^+^ cells were markedly elevated in GC samples compared to adjacent tissues (both *p* < 0.001; **Figures 5A, B**). No notable variation was found in the count of FAP^+^CPXM2^+^ cells (*p* = 0.111; **Figure 5C**), but their proportion was lower in GC (*p* = 0.013; **Figure 5D**). The proportion and count of FAP^+^CPXM2^-^CAV1^+^ cells were notably elevated in GC (both *p* < 0.001; **Figures 5E, F**).

### 3.4 Correlation of FAP, CAV1 and CPXM2 expression in GC tissues

**Figure 6:**
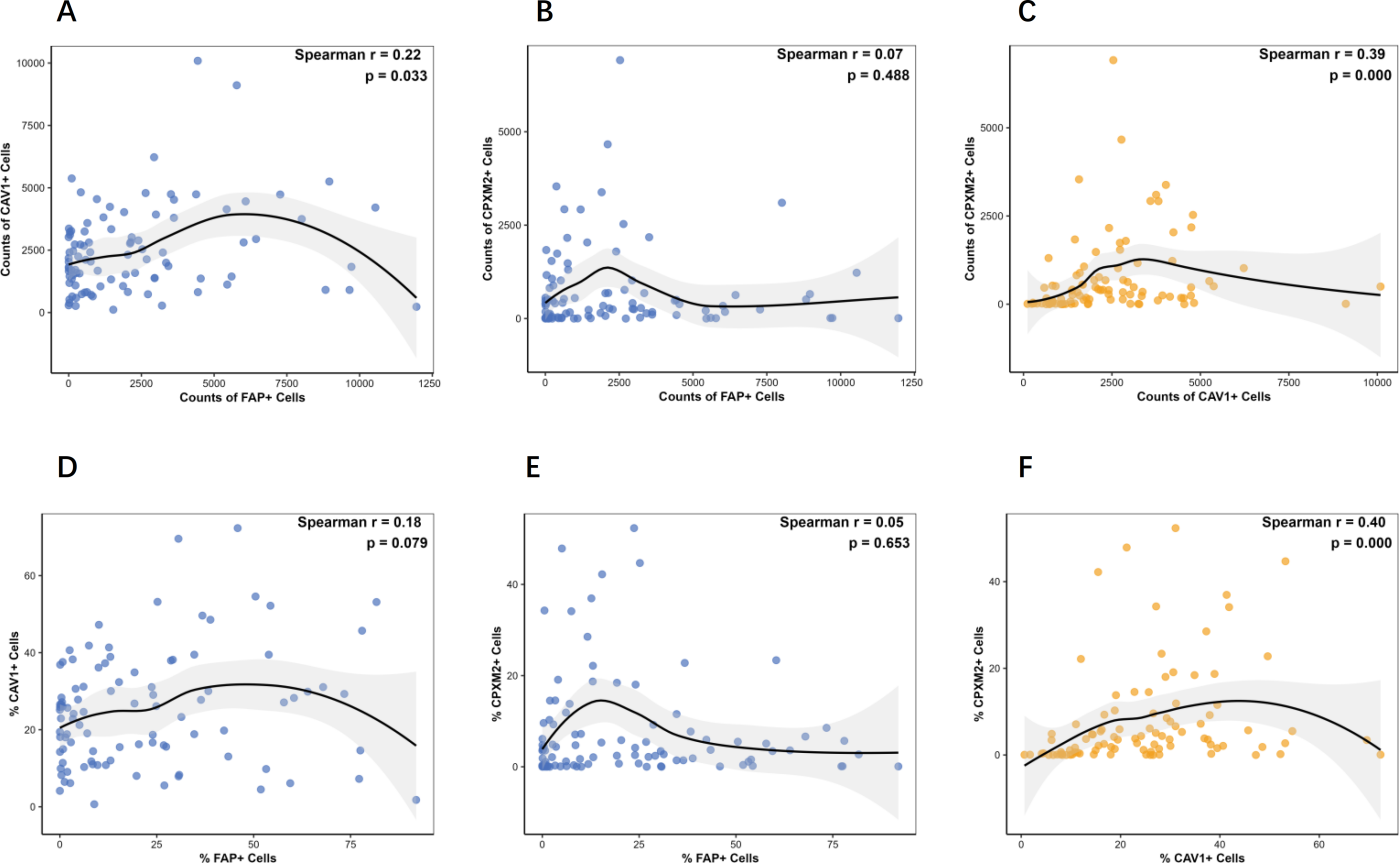
Correlation analysis of the counts and proportion of positive cells in GC. The correlation between the counts of FAP, CAV1 and CPXM2 positive cells **(A-C)** and the correlation between the proportion of FAP, CAV1 and CPXM2 positive cells in GC tissue samples **(D-F)**. Statistical tests were analyzed using Spearman’s correlation.

We analyzed correlations among FAP^+^, CAV1^+^, and CPXM2^+^ cell counts in gastric tissues (**Figure 6**). In GC samples, FAP^+^ cell counts positively correlated with CAV1^+^ cells (r = 0.22, *p* = 0.033; **Figure 6A**), but not with CPXM2^+^ cells (*p* > 0.05; **Figure 6B**). A strong positive correlation was observed between CAV1^+^ and CPXM2^+^ cell counts (r = 0.39, *p* < 0.001; **Figure 6C**). We also evaluated correlations among cellular percentages. No significant associations were found between the proportion of FAP^+^ cells and either CPXM2^+^ or CAV1^+^ cells in GC tissues (both *p* > 0.05; **Figures 6D, E**). In contrast, the percentages of CAV1^+^ and CPXM2^+^ cells showed a positive correlation (r = 0.40, *p* < 0.001; **Figure 6F**).

### 3.5 Correlation of FAP, CAV1 and CPXM2 expression with clinicopathological factors in GC tissues

We categorized 96 GC tissue samples into low and elevated expression level groups based on the proportion of FAP^+^, CAV1^+^, and CPXM2^+^ cells. Cutoff values were computed through the application of the survminer R package. We examined the relationship between the expression levels of these markers and the clinicopathological characteristics of GC patients, as detailed in **Table 1**. Chi-squared tests revealed significant associations between FAP expression and both AJCC stage (*p* = 0.017) and tumor invasion depth (*p* = 0.006). CPXM2 expression correlated with tumor location (p = 0.048), while CAV1 expression showed no significant relationship with any clinicopathological variables. Further analysis indicated that the percentage of CPXM2^+^CK^+^ cells correlated strongly with patient’s age (*p* < 0.001, **Table 2**).

**Table 1:** Clinical characteristics of GC patients.

Variables		n	FAP^+^ cells (%)		*P* value	CAV1^+^ cells (%)		*P* value	CPXM2^+^cells (%)		*P* value
			Low(11) High (85)			Low(30) High (66)			Low(51) High (45)		
Gender	female	23	1	22	0.288	9	14	0.350	12	11	0.917
	male	73	10	63		21	52		39	34	
Age	<65	64	9	55	0.327	19	45	0.640	36	28	0.386
	≥65	32	2	30		11	21		15	17	
Tumor size(cm)	<5	69	9	60	0.723	19	50	0.209	37	32	0.876
	≥5	27	2	25		11	16		14	13	
Grade	G1+G2	52	5	47	0.538	16	36	0.852	27	25	0.705
	G3+G4	43	6	37		14	29		24	19	
AJCC atage	I+II	46	9	37	**0.017**	12	34	0.295	22	24	0.318
	III+IV	50	2	48		18	32		29	21	
T stage	T1+T2	25	7	18	**0.006**	4	21	0.056	11	14	0.288
	T3+T4	71	4	67		26	45		40	31	
N stage	N0	28	3	25	1.000	5	23	0.069	11	17	0.081
	N1+	68	8	60		25	43		40	28	
M stage	M0	83	11	72	0.351	25	58	0.536	43	40	0.513
	M1	13	0	13		5	8		8	5	
Grade of differentiation	poor	48	5	43	0.605	15	33	0.916	27	21	0.479
	Well or Moderate	43	6	37		13	30		21	22	
Tumor location	Cardia/Proximal	15	0	15	0.054	3	12	0.379	10	5	**0.048**
	Fundus/Body	16	0	16		7	9		12	4	
	Antrum/Distal	65	11	54		20	45		29	36	

The P-values with statistical differences were highlighted in bold.

**Table 2:** Clinical characteristics of GC patients.

Variables		CPXM2^+^CK^+^ cells (%)		*p* value
		Low (41)	High (55)	
Gender	female	8	15	0.378
	male	33	40	
Age	<65	12	52	**<0.001**
	≥65	29	3	
Tumor size(cm)	<5	29	40	0.830
	≥5	12	15	
Grade	G1+G2	22	30	0.854
	G3+G4	19	24	
AJCC atage	I+II	19	27	0.790
	III+IV	22	28	
T stage	T1+T2	8	17	0.208
	T3+T4	33	38	
N stage	N0	8	20	0.072
	N1+	33	35	
M stage	M0	35	48	0.787
	M1	6	7	
Grade of differentiation	poor	23	25	0.208
	Well or Moderate	15	28	
Tumor location	Cardia/Proximal	6	9	0.071
	Fundus/Body	11	5	
	Antrum/Distal	24	41	

We also evaluated correlations involving CAV1 and CPXM2 co-expression in CAFs with clinical parameters (**Table 3**). The proportions of FAP^+^CAV1^+^ and FAP^+^CPXM2^-^CAV1^+^ cells demonstrated significant correlations with AJCC stage (*p* = 0.006 and *p* = 0.009, respectively). Furthermore, FAP^+^CAV1^+^ cell abundance correlated with invasion depth (*p* = 0.006), and FAP^+^CPXM2^-^CAV1^+^ cells were linked to tumor location (*p* = 0.031).

**Table 3:** Clinical characteristics of GC patients.

Variables		n	FAP^+^ CAV1^+^细胞(%)		*P* value	FAP^+^CPXM2^-^CAV1^+^细胞(%)		*P* value
			Low (20) High (76)			Low (17) High (79)		
Gender	female	23	4	19	0.774	3	20	0.755
	male	73	16	57		14	59	
Age	<65	64	15	49	0.374	11	53	0.850
	≥65	32	5	27		6	26	
Tumor size(cm)	<5	69	17	52	0.142	14	55	0.381
	≥5	27	3	24		3	24	
Grade	G1+G2	52	10	42	0.632	8	44	0.483
	G3+G4	43	10	33		9	34	
AJCC atage	I+II	46	15	31	**0.006**	13	33	**0.009**
	III+IV	50	5	45		4	46	
T stage	T1+T2	25	10	15	**0.006**	8	17	0.063
	T3+T4	71	10	61		9	62	
N stage	N0	28	4	24	0.311	4	24	0.770
	N1+	68	16	52		13	55	
M stage	M0	83	19	64	0.290	17	66	0.116
	M1	13	1	12		0	13	
Grade of differentiation	poor	48	10	38	0.991	9	39	0.757
	Well or Moderate	43	9	34		7	36	
Tumor location	Cardia/Proximal	15	1	14	0.357	0	15	**0.031**
	Fundus/Body	16	3	13		1	15	
	Antrum/Distal	65	16	49		16	49	

The P-values with statistical differences were highlighted in bold.

### 3.6 Prognostic value of FAP, CAV1 and CPXM2 in GC tissues

**Figure 7:**
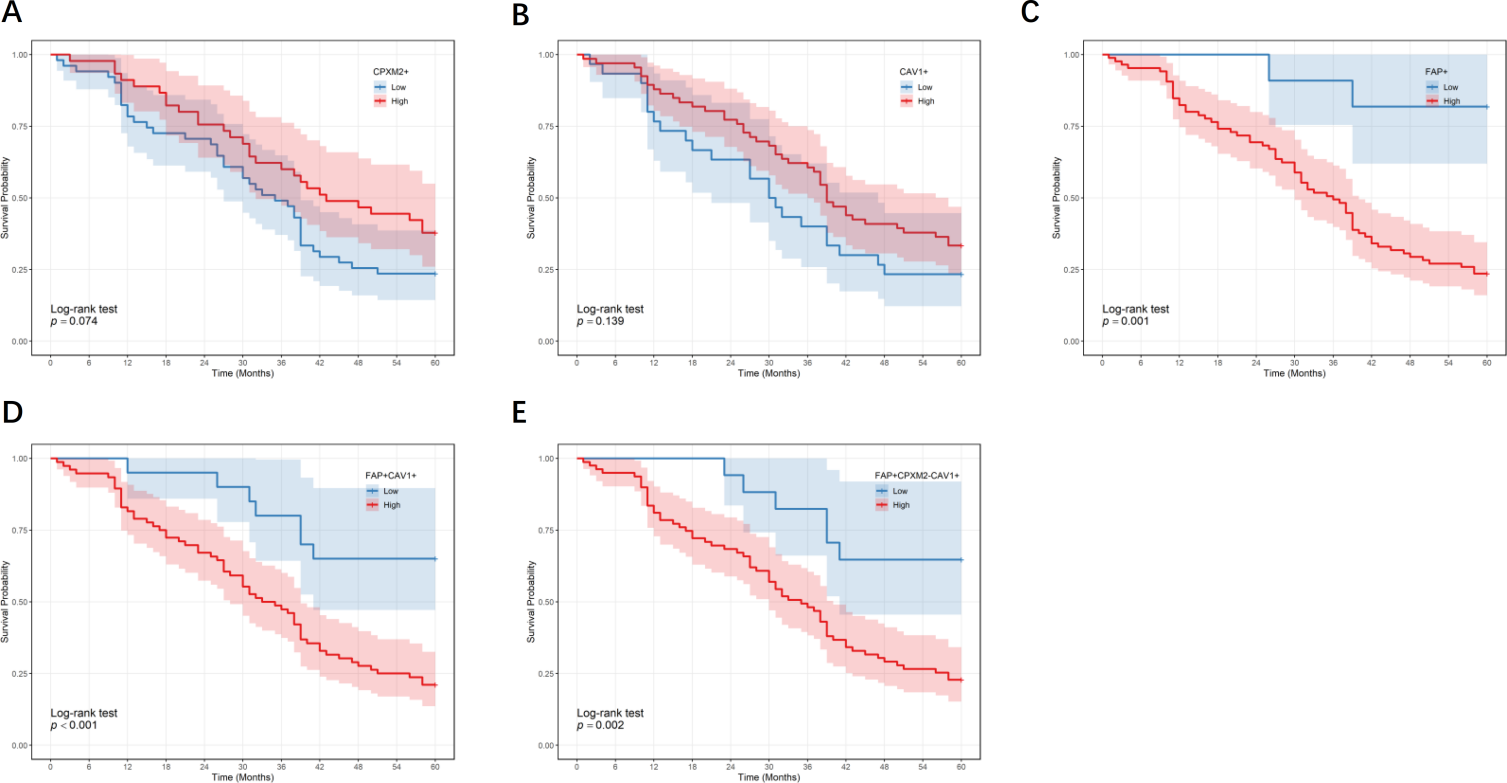
Kaplan-Meier analysis in GC. The prognosis of GC patients based on the percentage of CPXM2^+^ cells **(A)**, CAV1^+^ cells **(B)**, FAP^+^ cells **(C)**, FAP^+^CAV1^+^cells **(D)**, FAP^+^CPXM2^-^CAV1^+^ cells **(E)**.

These data demonstrated that the percentage of CPXM2^+^ cells (**Figure 7A**, *p* = 0.074) and CAV1^+^ cells (**Figure 7B**, *p* = 0.139) on patient prognosis was not significant, In addition, a higher percentage of FAP^+^ cells (**Figure 7C**, *p* = 0.001), FAP^+^CAV1^+^ cells (**Figure 7D**, *p* < 0.001) and FAP^+^CPXM2^-^CAV1^+^ cells (**Figure 7E**, *p* = 0.002) was markedly linked to a shorter OS. Our findings indicate that the percentage of FAP^+^, FAP^+^CAV1^+^ and FAP^+^CPXM2^-^CAV1^+^ cells can serve as significant prognostic indicators in GC.

Survival analysis on a univariate level pinpointed several significant predictors for OS, including tumor size (*p* = 0.013), TNM stage (*p* < 0.001), tumor invasion depth (*p* < 0.001), lymphatic metastasis (*p* = 0.033), distant metastasis (*p* < 0.001), a higher percentage of FAP^+^ cells (*p* = 0.007), FAP^+^CAV1^+^ cells (*p* = 0.002) and FAP^+^CPXM2^-^CAV1^+^cells (*p* = 0.005) were associated with poorer OS; multivariate Cox regression revealed that a higher percentage of TNM stage (*p* = 0.007), distant metastasis (*p* < 0.001) and FAP^+^CAV1^+^ cells (*p* = 0.017) stood out as distinct risk factors influencing patient prognosis (**Table 4**).

**Table 4:** Univariate and multivariate cox regression analysis of GC.

Variable	n	Univariate analysis			Multivariate analysis		
		HR	95%	*P*	HR	95%	*P*
Grade		1.001	0.617-1.624	0.997			
G1+G2	52						
G3+G4	43						
Gender		1.044	0.602-1.811	0.878			
male	73						
female	23						
Age		1.551	0.942-2.553	0.085			
<65	64						
≥65	32						
Tumor size(cm)		1.917	1.146-3.206	**0.013**			
<5	69						
≥5	27						
Tumor location							
Cardia/Proximal	15			0.454			
Fundus/Body	16	0.835	0.408-1.707	0.621			
Antrum/Distal	65	1.209	0.640-2.284	0.558			
TNM stage		3.171	1.745-5.761	**<0.001**	2.338	1.258-4.345	**0.007**
I+II	32						
III+IV	64						
T stage		3.390	1.718-6.690	**<0.001**			
T1+T2	25						
T3+T4	71						
N stage		1.847	1.050-3.250	**0.033**			
N0	28						
N1+	68						
M stage		7.960	3.940-16.084	**<0.001**	5.393	2.652-10.968	**<0.001**
M0	83						
M1	13						
Grade of differentiation		0.634	0.382-1.052	0.078			
poor	48						
Well or Moderate	43						
CAV1		0.687	0.415-1.140	0.146			
low	30						
high	66						
FAP		6.982	1.706-28.581	**0.007**			
low	11						
high	85						
CPXM2		0.647	0.398-1.054	0.080			
low	51						
high	45						
FAP^+^CAV1^+^		3.504	1.597-7.685	**0.002**	2.640	1.188-5.866	**0.017**
low	20						
high	76						
FAP^+^CPXM2^-^CAV1^+^		3.358	1.449-7.785	**0.005**			
low	17						
high	79						

The P-values with statistical differences were highlighted in bold.

## 4 Discussion

In the current study, our initial objective is to examine FAP and α-SMA, common CAF markers, in GC immunostaining. In line with prior research (20, 21), our findings further revealed ubiquitous α-SMA expression throughout the GC tumor microenvironment, localizing to CAFs, smooth muscle cells, and pericytes. This broad distribution precludes its use as a specific CAF marker. In contrast, FAP expression was strictly confined to fibroblasts, supporting its utility as a more reliable and specific CAF marker. We assessed the expression of FAP by counting the number and percentage of positive cells in the tissue. Analysis demonstrated significantly elevated FAP levels in CAFs compared to fibroblasts in precancerous tissues. Furthermore, FAP expression was positively correlated with tumor invasion depth and AJCC cancer stage; higher FAP expression is associated with shorter survival, further validating the results of previous reports (21, 22). These observations demonstrated that FAP is not only a robust stromal biomarker but also a promising target for stromal-directed therapies.

The role of CAV1 in cancer cells is still controversial; however, CAV1 high GC cells reveal a survival advantage via endocytosis to utilize extracellular protein (23). In lung cancer, patients not only with CAV1-positive cancer cells but also with CAV1-positive CAFs show poor prognosis (24). Furthermore, CAV1 expressed CAFs contribute to tumor invasion, resulting in poor prognosis in pancreatic cancer patients (25). In this study, we demonstrated that CAV1 expression was markedly increased in tumor samples compared to precancerous ones. However, no notable association was detected between CAV1 levels and clinicopathological features or patient prognosis. Although CAV1 levels did not exhibit a direct correlation with patient survival or standard clinicopathological variables, this stromal-specific upregulation suggests a potential role for CAV1 in modulating the tumor microenvironment, possibly through regulation of stromal epithelial crosstalk or CAFs activation. The dualistic nature of CAV1, which can function as either an oncogene or a tumor suppressor, has been the subject of extensive debate (11), and our data suggest that CAV1 is predominantly expressed in the stromal cells of gastric cancer. Nevertheless, the specific stromal cell types in which it is expressed and the role it plays still await further investigation.

Previous studies have linked CPXM2 to developmental disorders (12, 13) and Alzheimer’s disease (14), yet its potential role in tumorigenesis or cancer progression remains unclear. In the current study, we observed reduced CPXM2 expression in gastric tumor specimens compared with adjacent tissues. Moreover, the expression of CPXM2 was significantly correlated with tumor location. Using the CK marker to differentiate epithelial from stromal compartments, we found that CPXM2 expression levels did not significantly differ between epithelial and stromal components in GC tissues. When compared with adjacent noncancerous tissues, no significant variation in CPXM2 expression was detected in stromal cells. In contrast, CPXM2 expression in epithelial cells was markedly reduced compared to adjacent tissues and exhibited a correlation with patient age. Niu et al. previously reported that elevated expression of CPXM2 in GC tissues promotes tumor progression and is linked to a poor prognosis for patients (16). Our research results suggest that its function may be more complex. It is conceivable that CPXM2 plays differing roles across cancer types or molecular subtypes. Further mechanistic studies are essential to clarify whether CPXM2 acts as a tumor suppressor in GC.

Interestingly, CAV1 and CPXM2 show a significant positive correlation in GC tissues. The correlation analysis of CAV1 and CPXM2 on the GEPIA online website further confirms our findings (**Supplementary Figure 1**). Although this correlation does not imply causation, this novel association warrants further investigation to uncover any mechanistic links between CAV1 and CPXM2 in GC biology.

CAFs constitute the predominant cell population within the tumor stroma. We employed FAP, a specific marker for CAFs, to assess the expression of CAV1 and CPXM2 in CAFs and their prognostic significance. The findings indicated that the expression of CPXM2 in CAFs did not exhibit a significant difference between GC tissues and adjacent non-neoplastic tissues. However, CAV1 was notably elevated in GC tissues, and its high expression was correlated with a poor prognosis. Based on the finding that CPXM2 expression is decreased in GC tissues and may play a tumor suppressor role, we also analyzed the expression of the dual indicators CAV1 and CPXM2 in CAFs. The proportion of CPXM2^-^CAV1^+^ CAFs is significantly elevated in GC tissues, and a higher prevalence is correlated with poorer clinical outcomes. Suggesting that loss of CPXM2 in CAV1-expressing CAFs may further enhance their pro-tumorigenic functions.

Substantial recent evidence demonstrates that depleting FAP^+^CAFs enhances anti-tumor immunity (26–30). Chen et al. characterized FAP^+^CAFs as a major stromal component linked to immunosuppression and unfavorable prognosis. Functionally, these cells secrete abundant fibronectin 1 (FN1), which binds integrin α5β1 on macrophages, activating the FAK-AKT-STAT3 signaling pathway and promoting their polarization into an immunosuppressive M2-like phenotype (31). We observed that CAV1 was predominantly expressed in the tumor stroma, with its expression in FAP^+^CAFs being significantly upregulated in tumor tissues. To investigate the potential involvement of CAV1 in CAFs in the context of immunotherapy, we utilized the online database GEPIA to analyze CAV1 expression across various immune cell types. Significant alterations in CAV1 levels were detected in B cells, CD8^+^ T cells, and macrophages in GC compared to normal tissues, implying that stromal CAV1 may influence immune cell activity within the tumor microenvironment (**Supplementary Figure 2**).

This study also has some limitations. It was conducted in a single center with a limited sample size. In future studies, we will increase the sample size and employ single-cell sequencing and spatial transcriptomic technologies to further investigate the functional roles of CAV1 and CPXM2 in GC, providing new therapeutic targets for the treatment of GC.

## 5 Conclusions

Overall, in this current study, we evaluated the expression of FAP, CAV1, CPXM2, and CK in GC and adjacent tissues samples using mIF. Elevated expressions of CAV1^+^ and CPXM2^-^CAVI^+^ in CAFs were linked to a poor prognosis in GC patients. Moreover, CAV1 and CPXM2 warrant further investigation as potential biomarkers and candidates for mechanistic studies exploring their roles in GC progression, particularly in cases with a high prevalence of CAFs.

## Data Availability

The raw data supporting the conclusions of this article will be made available by the authors, without undue reservation.
