# Supplementary material for: Analysis of the expression of FAP, Caveolin1, and CPXM2 and prognostic significance in gastric cancer
Source: Front Oncol. 2026 Apr 13;16:1716536. doi: 10.3389/fonc.2026.1716536 (PMC13110993; doi:10.3389/fonc.2026.1716536)
Supplement: Supplementary file 1 [file DataSheet1.docx]

Supplementary Material

## Supplementary Figures


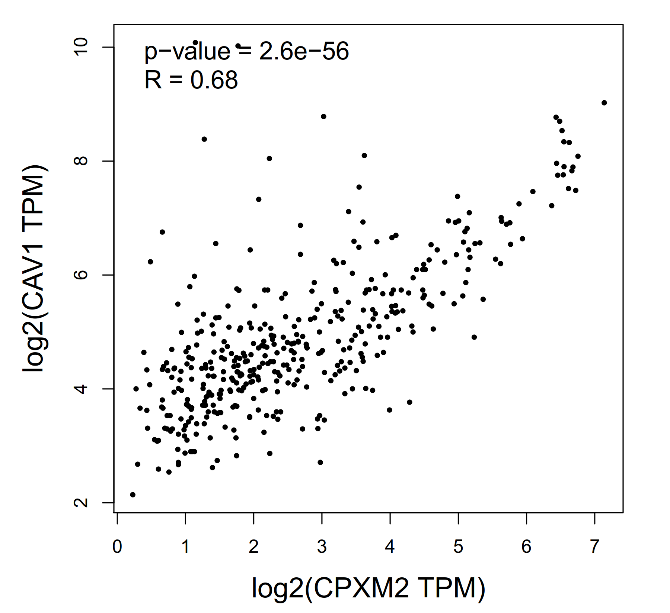


**Supplementary Figure 1.** The figure legends are required to have the same font as the main text, 12 point normal Times New Roman, single spaced. Please use a single paragraph for each legend and prepare the figures keeping in mind the PDF layout.

A


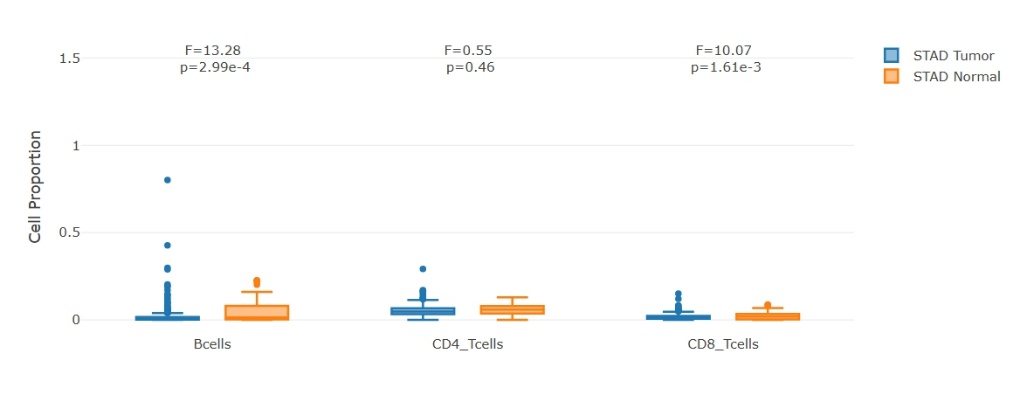


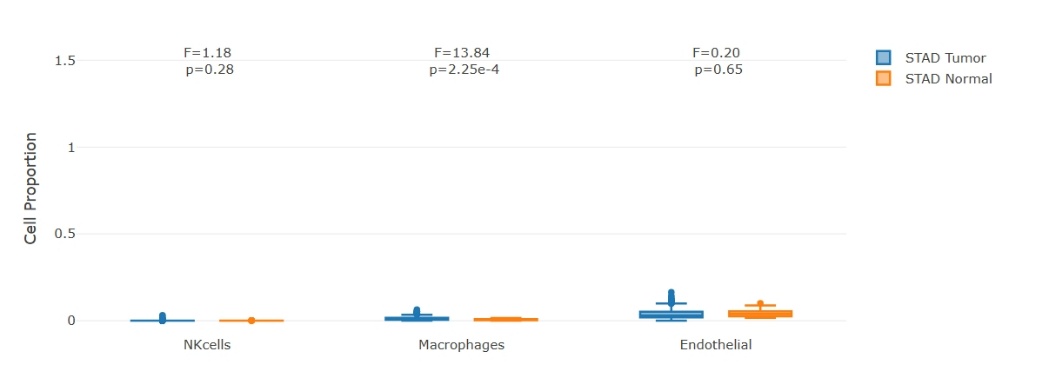
B

**Supplementary Figure 2:** Predicting the expression of CAV1 in stromal cells of gastric tissues. Boxplots were used to visualize the expression of log (TPM+1) CAV1 in each cell type selected, by EPIC (A-B).
